# Supplementary material for: AffyMAPSDetector: a software tool to characterize Affymetrix GeneChip™ expression arrays with respect to SNPs
Source: BMC Bioinformatics. 2007 Jul 30;8:276. doi: 10.1186/1471-2105-8-276 (PMC1959249; doi:10.1186/1471-2105-8-276)
Supplement: Additional file 13 — AffyMAPSDetector v1 source code. AffyMAPSDetector source code. [file 1471-2105-8-276-S13.zip › AffyMAPSDetector_v1_src/doc/affychip/snpdetector/model/SnpMatcher.html]

SnpMatcher


|  |  |  |  |
| --- | --- | --- | --- |
| **Overview** \| **Package** \| **Class** \| **Use** \| **Tree** \| **Deprecated** \| **Index** \| **Help** | | |  |
| **PREV CLASS**   **NEXT CLASS** | **FRAMES**    **NO FRAMES**     **All Classes** |  |  |
| SUMMARY: NESTED \| FIELD \| CONSTR \| METHOD | DETAIL: FIELD \| CONSTR \| METHOD |  |  |


---


## affychip.snpdetector.model Class SnpMatcher

```
java.lang.Object
  affychip.snpdetector.model.SnpMatcher
```

---

public class **SnpMatcher** extends java.lang.Object

Title: Probe SNP Detector

Description:

This program does the following:  
1. It parses probe-sequence file (HG-U95Av2\_probe.fasta - entered as 1st commandline argument).  
2. It parses gene-info file (HG\_U95Av2\_Array\_Gene\_Info.xls - entered as 2nd commandline argument).  
3. From gene-info file, it is determined that if locus-link is defined for a
gene. If locus-link is not defined, then this gene-info is written out to
HG\_U95Av2\_Gene\_Info(No\_Locus\_Link).xls file. If locus-link is defined
then gene-id (GenBank Accession number) and locus-link-id are extracted and
NCBI SNP database is querried for possible SNPs. If SNPs are found then the
gene-info is enhanced (SNP results) and written out to HG\_U95Av2\_Gene\_Info(Has\_SNPs).xls
otherwise gene-info is written out to HG\_U95Av2\_Gene\_Info(No\_SNPs).xls. In addtion,
this program also generates two more tab deliminted files: "gene info extracted from web" and
"snp info extracted from web" (gene identifier being key of each record in these files).

Copyright: Copyright (c) 2004

Company: George Mason University

**Version:**
:   1.1

**Author:**
:   Sunita Kumari

---

|  |  |
| --- | --- |
| **Nested Class Summary** | |
| `class` | `SnpMatcher.FileClosingShutdownHook`             Shudown hook that would ensure that if the program terminates abnormally then all opened data files, to which data is written, are properly closed without loosing any data that has not yet been commited to disk. |


|  |  |
| --- | --- |
| **Field Summary** | |
| `static java.lang.String[]` | `ADDITIONAL_GENE_HEADERS` |
| `static org.apache.log4j.Logger` | `logger` |


**Constructor Summary**

`SnpMatcher(DataState ds)`


|  |  |
| --- | --- |
| **Method Summary** | |
| `void` | `addStatusEventListener(StatusEventListener sel)` |
| `void` | `fireStatusUpdateEvent(StatusEvent e)` |
| `boolean` | `isHaltExecution()` |
| `boolean` | `isStopExecution()` |
| `void` | `removeStatusEventListener(StatusEventListener sel)` |
| `void` | `setHaltExecution(boolean halt)` |
| `void` | `setStopExecution(boolean stop)` |
| `void` | `startMatch()` |

**Methods inherited from class java.lang.Object**

`clone, equals, finalize, getClass, hashCode, notify, notifyAll, toString, wait, wait, wait`

**Field Detail**

### logger

```
public static org.apache.log4j.Logger logger
```

---


### ADDITIONAL\_GENE\_HEADERS

```
public static final java.lang.String[] ADDITIONAL_GENE_HEADERS
```


**Constructor Detail**

### SnpMatcher

```
public SnpMatcher(DataState ds)
```


**Method Detail**

### setStopExecution

```
public void setStopExecution(boolean stop)
```

---


### isStopExecution

```
public boolean isStopExecution()
```

---


### isHaltExecution

```
public boolean isHaltExecution()
```

---


### setHaltExecution

```
public void setHaltExecution(boolean halt)
```

---


### addStatusEventListener

```
public void addStatusEventListener(StatusEventListener sel)
```

---


### removeStatusEventListener

```
public void removeStatusEventListener(StatusEventListener sel)
```

---


### fireStatusUpdateEvent

```
public void fireStatusUpdateEvent(StatusEvent e)
```

---


### startMatch

```
public void startMatch()
```


---


|  |  |  |  |
| --- | --- | --- | --- |
| **Overview** \| **Package** \| **Class** \| **Use** \| **Tree** \| **Deprecated** \| **Index** \| **Help** | | |  |
| **PREV CLASS**   **NEXT CLASS** | **FRAMES**    **NO FRAMES**     **All Classes** |  |  |
| SUMMARY: NESTED \| FIELD \| CONSTR \| METHOD | DETAIL: FIELD \| CONSTR \| METHOD |  |  |


---
